# Short Intracortical Inhibition During Voluntary Movement Reveals Persistent Impairment Post-stroke

**DOI:** 10.3389/fneur.2018.01105

**Published:** 2019-01-04

**Authors:** Qian Ding, William J. Triggs, Sahana M. Kamath, Carolynn Patten

**Affiliations:** ^1^Biomechanics, Rehabilitation, and Integrative Neuroscience Lab, Department of Physical Medicine and Rehabilitation, School of Medicine, University of California, Davis, Sacramento, CA, United States; ^2^Rehabilitation Science PhD Program, University of Florida, Gainesville, FL, United States; ^3^Brain Rehabilitation Research Center, Malcom Randall VA Medical Center, Gainesville, FL, United States; ^4^Department of Neurology, University of Florida, Gainesville, FL, United States

**Keywords:** GABA_A_, inhibition, motor control, SICI, stroke

## Abstract

**Objective:** Short intracortical inhibition (SICI) is a GABA_A_-mediated phenomenon, argued to mediate selective muscle activation during coordinated motor activity. Markedly reduced SICI has been observed in the acute period following stroke and, based on findings in animal models, it has been posited this disinhibitory phenomenon may facilitate neural plasticity and contribute to early motor recovery. However, it remains unresolved whether SICI normalizes over time, as part of the natural course of stroke recovery. Whether intracortical inhibition contributes to motor recovery in chronic stroke also remains unclear. Notably, SICI is typically measured at rest, which may not fully reveal its role in motor control. Here we investigated SICI at rest and during voluntary motor activity to determine: (1) whether GABA_A_-mediated inhibition recovers, and (2) how GABA_A_-mediated inhibition is related to motor function, in the chronic phase post-stroke.

**Methods:** We studied 16 chronic stroke survivors (age: 64.6 ± 9.3 years; chronicity: 74.3 ± 52.9 months) and 12 age-matched healthy controls. We used paired-pulse transcranial magnetic stimulation (TMS) to induce SICI during three conditions: rest, submaximal grip, and performance of box-and-blocks. Upper-extremity Fugl-Meyer Assessment and Box-and-Blocks tests were used to evaluate motor impairment in stroke survivors and manual dexterity in all participants, respectively.

**Results:** At rest, SICI revealed no differences between ipsilesional and contralesional hemispheres of either cortical or subcortical stroke survivors, or healthy controls (*P*'s > 0.05). During box-and-blocks, however, ipsilesional hemisphere SICI was significantly reduced (*P* = 0.025), especially following cortical stroke (*P* < 0.001). SICI in the ipsilesional hemisphere during box-and-blocks task was significantly related to paretic hand dexterity (*r* = 0.56, *P* = 0.039) and motor impairment (*r* = 0.56, *P* = 0.037).

**Conclusions:** SICI during motor activity, but not rest, reveals persistent impairment in chronic stroke survivors indicating that inhibitory brain circuits responsible for motor coordination do not fully normalize as part of the natural history of stroke recovery. Observation that reduced SICI (i.e., disinhibition) is associated with greater motor impairment and worse dexterity in chronic hemiparetic individuals suggests the response considered to promote neuroplasticity and recovery in the acute phase could be maladaptive in the chronic phase post-stroke.

## Introduction

GABAergic inhibitory brain circuits are important to motor control (1–6). These inhibitory circuits are suggested to prevent co-activation of separate motor cortical regions in animal models (1) and are implicated in selective muscle activation during dexterous motor tasks in humans (1–6).

Paradoxically, reduced GABAergic activity, or disinhibition, is considered relevant to early motor recovery following stroke (7–10). This argument stems from observations in a mouse model of acute stroke that excessive GABA_A_-mediated tonic inhibition is reduced by blockage of extrasynaptic GABA_A_ receptors (11). Functional recovery of forelimb and hindlimb motor control is associated with this reduced inhibition (11). It is thus reasoned that disinhibition of GABA_A_ activity enhances neuroplasticity and promotes functional reorganization of perilesional tissue contributing to functional recovery (12). As a result, reduced GABAergic activity, or disinhibition, is also believed to be relevant to early motor recovery following stroke in humans (7–10). However, due to differences in both the underlying biology and measurement of GABAergic activity, it remains unclear how well results from animals models can be generalized to humans.

The role of cortical disinhibition in the ipsilesional hemisphere (IH) becomes even less clear in the chronic phase post-stroke when an alternative motor network has become established (8, 13–15). Current views on stroke rehabilitation emphasize means to increase IH cortical excitability [i.e., non-invasive brain stimulation (NIBS)] in both acute and chronic stroke survivors with expectation this approach will improve upper limb motor function (16–20). Recent meta-analyses of NIBS clinical trials report unsatisfactory outcomes; while IH cortical excitability can be increased, it does not appear to be effectively translated to functional improvements in the paretic arm (21, 22). However, these studies focus on cortical excitability without consideration of the role of intracortical inhibitory circuits in motor control. Of note, Marconi et al. (14) found intervention-related improvements in motor function in chronic stroke survivors correspond with increased intracortical inhibition. Similar results have been reported by Liepert et al. (13). Together, these observations suggest enhancement of intracortical inhibitory activity in the chronic phase post-stroke may contribute to remodeling of the residual motor network (14) and promote motor recovery more effectively than a further loss of inhibition (13). Given limited evidence to support these suggestions, the role of intracortical inhibition in motor recovery in chronic stroke remains unclear.

Short intracortical inhibition (SICI), induced using paired-pulse transcranial magnetic stimulation (TMS), reflects activity of GABA_A_-mediated inhibitory circuits (23–25). The literature suggests SICI is reduced in the IH acutely (i.e., within a month) post-stroke (7, 8, 10, 26), but may return to normal levels chronically (i.e., >6 months) (15, 27, 28). Beyond these fundamental observations, the current literature discussing SICI post-stroke lacks a common thread. For example, differences in the magnitude of IH SICI have been reported between cortical and subcortical stroke by some (13, 27) but not all (8, 10, 26, 28) investigators. Inconsistencies are also found in the relationship between paretic hand motor function and IH SICI. For example, Honaga et al. (28) reported that motor function and SICI were inversely related in chronic stroke survivors (i.e., lower-functioning individuals tend to show disinhibition), while Ferreiro de Andrade et al. (29) recently reported the opposite. Still other studies report no correlation between SICI and motor function in chronic stroke (8, 13, 27). The influence of lesion location on SICI is also unclear. For example, SICI has been found to be more disrupted in the early phase following cortical vs. subcortical stroke (27, 30). However, it has also been reported that lesion location does not influence IH SICI_rest_ in chronic stroke (8, 10, 26, 28).

Such inconsistencies in detecting SICI may stem from multiple confounding factors. For one, SICI is usually measured at rest, particularly in stroke survivors. A few studies have measured SICI during motor preparation in stroke surviors (31, 32). However, since GABAergic circuits are implicated in motor function, SICI measured at rest (SICI_rest_), or prior to movement, may not elicit the same phenomenon as SICI measured during production of motor activity (SICI_active_). Another key factor, motor-evoked potential (MEP) size, is more variable at rest than during voluntary muscle contraction (33). To our knowledge, no published study has measured SICI during muscle contraction or motor activity (i.e., SICI_active_) in stroke survivors.

Here we investigated the relationship between SICI and motor function in the chronic phase post-stroke to determine whether IH SICI is normalized as part of the natural history of recovery. We studied individuals with hyper-chronic stroke sequelae at rest and during active motor tasks to investigate: (1) whether SICI is normalized, (2) whether recovery of SICI differs between cortical and subcortical stroke survivors, and (3) the relationship of IH SICI during motor activity (SICI_active_), relative to healthy controls. We anticipated IH SICI_active_ would be reduced in stroke survivors, especially following cortical stroke. These results have potential implications for both understanding the process of motor recovery and identifying rehabilitation strategies to promote recovery following stroke.

## Methods

### Subjects

We studied sixteen chronic stroke survivors and twelve age-matched healthy controls. Stroke survivors meeting the following criteria were included: (1) evidence of a single, monohemispheric stroke (with confirmatory neuroimaging) ≥6 months prior to enrollment with (2) nominal ability to form and release a power grip and transfer small objects as required by the Box and Blocks Test (BBT) (34). Healthy, age-matched adults with no history of stroke or chronic neurological impairment were studied as reference control subjects. All participants were screened for eligibility to receive TMS (35) and excluded if: using medications that reduce seizure threshold; pregnant; or any implanted device or metal that might be affected by the magnetic field generated by TMS was present. Additional study exclusion criteria were: presence of cognitive impairment as defined by inability to comprehend and follow three step commands; corrected vision <20/20; or history of seizure disorder. Stroke survivors were classified as cortical or subcortical stroke if the lesions involved cortical areas in any vessel distribution or affected only subcortical areas, respectively. Demographic characteristics are reported in Tables 1, 2.

**Table 1:** Participants demographic and clinical characteristics.

	**Age, years**	**Sex**	**Paretic side**	**Handedness (premorbid in stroke)**	**Months after stroke onset**	**FMA** **(0–66)**	**MAS** **(0–28)**	**BBT (P or ND arm)**	**MoCA** **(0–30)**
	**Mean** **±*****SD*** **(range)**	**Male/Female**	**Right/Left**	**Right/Left**	**Mean** **±*****SD*** **(range)**	**Mean** **±*****SD*** **(range)**	**Mean** **±*****SD*** **(range)**	**Mean** **±*****SD*** **(range)**	**Mean** **±*****SD*** **(range)**
Cortical stroke (*n* = 8)	65.1 ± 11.1 (49–81)	7/1	5/3	8/0	88.3 ± 57.8 (6–170)	58.0 ± 10.1 (38–66)	3.8 ± 8.0 (0–23)	37.6 ± 11.4 (21–49)	24.8 ± 5.7 (16–30)
Subcortical stroke (*n* = 8)	62.6 ± 7.7 (53–77)	7/1	2/6	8/0	60.3 ± 46.9 (7–175)	55.6 ± 10.1 (38–66)	4.3 ± 7.4 (0–21)	33.8 ± 10.2 (16–47)	27.3 ± 3.6 (21–30)
Healthy controls (*n* = 12)	60.6 ± 8.8 (51–80)	7/5	n/a	12/0	n/a	n/a	n/a	51.2 ± 11.4 (31–75)	27.7 ± 2.5 (22–30)

*UE FMA refers to upper-extremity component of the Fugl-Meyer Motor Function Assessment, indicating motor impairments in stroke survivors (36). MAS refers to modified Ashworth scale, indicating spasticity in stroke survivors (37). BBT refers to box and blocks test, measuring manual dexterity (34). MoCA refers to Montreal cognitive assessment. P arm refers to paretic arm in stroke survivors. ND arm refers to non-dominant arm in healthy controls. No differences in age were revealed between cortical, subcortical stroke and control groups. No difference in chronicity, UE FMA, MAS, BBT, or MoCA was revealed between cortical and subcortical stroke groups*.

**Table 2:** Participant characteristics.

**Subject number**	**Age (years)**	**Sex**	**Paretic hand**	**Chronicity (mos)**	**Type of stroke**	**Lesion location**	**UE FMA**
Stroke 01	53	M	R	28	Ischemic	Posterior internal capsule; subcortical	58
Stroke 02	64	M	R	170	Ischemic	Frontal lobe and posterior parietal lobe; cortical	49
Stroke 03	58	M	L	34	Ischemic	Temporal/parietal lobe; cortical	38
Stroke 04	77	M	L	34	Ischemic	Thalamus; subcortical	66
Stroke 05	62	M	L	150	Ischemic	Posterior internal capsule; subcortical	38
Stroke 06	63	F	L	93	Ischemic	Internal capsule; subcortical	66
Stroke 07	81	M	R	143	Hemorrhagic	Temporal/parietal lobe; cortical	66
Stroke 08	74	M	R	70	Ischemic	Parietal lobe and insula; cortical	58
Stroke 09	67	M	R	66	Hemorrhagic	Periventricular white matter, centrum semiovale; subcortical	62
Stroke 10	66	M	L	80	Ischemic	Putamen and periventricular white matter; subcortical	44
Stroke 11	59	M	L	24	Ischemic	Posterior internal capsule; subcortical	55
Stroke 12	70	F	L	47	Hemorrhagic	Parietal/temporal lobe; cortical	65
Stroke 13	54	M	L	7	Hemorrhagic	Putamen and periventricular white matter; subcortical	56
Stroke 14	49	M	L	127	Ischemic	Temporal/frontal/parietal lobe; cortical	66
Stroke 15	53	M	R	110	Ischemic	Frontal/temporal/parietal lobe; cortical	57
Stroke 16	72	M	R	6	Ischemic	Insular; cortical	65

*UE FMA refers to upper-extremity component of the Fugl-Meyer Motor Function Assessment. M refers to male, and F refers to female. L refers to left, and R refers to right*.

All study procedures were approved by University of Florida Health Science Center Institutional Review Board (IRB-01) and carried out in conformity with the standards of the Declaration of Helsinki. Prior to enrollment all participants provided written informed consent.

### Clinical Assessments

Motor impairment in stroke survivors was assessed using the upper extremity component of the Fugl-Meyer Motor Assessment (UE-FMA) (36) and the Modified Ashworth Scale (MAS) (37). All participants were assessed with the Edinburgh Handedness Inventory, to determine laterality (38), and the BBT (34), to assess manual dexterity. The Montreal Cognitive Assessment (MoCA) was also administered in all participants to characterize cognitive function (39).

### Force Measurements

We tested maximal voluntary isometric power grip (MVC) in both hands of stroke survivors and the non-dominant hand of healthy controls. Custom grip dynamometers instrumented with capacitive load cells (iLoad Mini MFD-200 & DQ-1000A, Loadstar Sensors, Fremont, California) were used to measure isometric power grip force in the “standard” position (40) with real-time force feedback displayed on a television screen (Samsung, TruSurround HD, Dolby Digital, 48 inches). Three MVC trials were interspersed with rest intervals (2 min); the peak value was carried forward as MVC for each hand.

### EMG Recordings

MEPs were collected by recording surface EMG from the first dorsal interosseus (FDI) using the Surface EMG for Non-Invasive Assessment of Muscles (SENIAM) guidelines for electrode placement (41). Participants were seated in a comfortable chair with the back and neck supported. EMG signals were sampled at 2 kHz using LabChart (Version 7 Pro, AD instruments, Colorado Springs, Colorado, U.S.A.) via a laboratory analog-to-digital interface (PowerLab 16/35, AD instruments, Colorado Springs, Colorado, U.S.A.). EMG data were written to disc for offline analysis.

### Transcranial Magnetic Stimulation (TMS)

TMS was performed using two Magstim stimulators connected by a Bi-stim module (Magstim 200^2^ & BiStim^2^, The Magstim Company Ltd, Dyfed, Wales, UK). TMS was applied over primary motor cortex using a figure-of-eight-shaped coil (70 mm diameter) positioned tangentially 45° from midline to induce a posterior-anterior current in the target hemisphere. Participants were asked to rest while determining the optimal scalp position for eliciting maximal responses in contralateral FDI (i.e., “hotspot”). Resting motor threshold (RMT) was determined experimentally as the lowest stimulation intensity that produced MEPs ≥50 μV in >50% of consecutive stimulations (42) during rest, and active motor threshold (AMT) as the lowest stimulation intensity that produced MEPs ≥100 μV in >50% of consecutive stimulations while gripping at 10% MVC (43). Neuronavigation (BrainSight, Version 2, Rogue Research Inc., Montreal, Quebec, Canada, 2006) was used to maintain coil position over the hotspot and monitor its stability. Coil positioning error was controlled at <5 mm displacement and <3° relative to target. Stimulations were delivered at ≤0.1 Hz.

SICI was induced using paired-pulse TMS [i.e., conditioning (S1)—test (S2) stimuli delivered at a fixed interstimulus interval (ISI)]. During study parameterization, ISIs were tested (range 2–6 ms, 0.5 ms increments, randomized order) to identify the ISI producing maximal inhibition for each subject and hemisphere (44, 45). In the rest condition S1 was set at stimulator output equal to 80% RMT (23); during active motor tasks S1 was set at 70% AMT (46). S2 was adjusted across tasks to the stimulator intensity producing an MEP between 0.5 and 1 mV peak-to-peak during task performance (46).

We defined “SICI non-responders” for cases where SICI_rest_ could not be induced using any ISI. Such atypical SICI_rest_ (i.e., inability to induce inhibition) has been reported among older adults (47, 48), thus to eliminate this potential confounding factor, “SICI non-responders” were excluded from further analysis. This exclusion involved three healthy control participants, both hemispheres of one individual (subcortical) and the contralesional hemispheres (CH) of three stroke survivors (two cortical, one subcortical).

### Task-Dependent SICI

SICI was induced in three motor conditions: at rest (SICI_rest_), during submaximal grip (SICI_grip_), and during box & blocks (B&B) (SICI_B&B_). At rest, the arm was positioned in 5–10° of shoulder flexion, 10–15° shoulder abduction, and 90° elbow flexion, with the forearm and wrist in neutral supported by an armrest. Participants were instructed to completely relax. EMG signals displayed on a computer screen were used to provide feedback and assist participants in keeping the arm and hand muscles quiet. During grip, participants produced constant submaximal (10% MVC) isometric power grip with force feedback displayed visually as a target zone (10 ± 2% MVC) within which the participant was instructed to maintain force. The standard arm position was maintained during gripping (40, 49) with an arm support. Prior to testing, participants practiced using visual feedback to maintain the force trace within the target zone. TMS was applied when the force trace was stable and maintained in the target zone. During SICI testing, participants gripped for up to 20 s; 3–4 stimulations were delivered during each trial. Note, the B&B task condition differs slightly from the BBT, referenced above, used for assessment of dexterity. During B&B, participants transferred blocks between halves of a divided box at their preferred pace. The box and blocks task involves repeated reach, grasp, transfer, and release of a standard object, thus is considered an assay of functional movement. Attainment of maximal thumb-index finger aperture during hand pre-shaping is recognized as an invariant characteristic of reach-to-grasp movements (50). Therefore, to assure all participants were stimulated at the same stage of movement, we applied TMS concurrently with acquisition of maximal finger-thumb aperture during the reach-to-grasp stage.

### Experimental Procedures

All procedures were conducted in a single session. TMS testing was performed in both hemispheres in stroke survivors and the non-dominant hemisphere in healthy controls. SICI testing followed TMS parameterization to determine RMT, AMT, S2, and ISI. In stroke survivors, IH and CH were tested in random order. Task order was randomized by subject; within each task conditioned and unconditioned stimuli (20 each) were block randomized (four stimuli per block).

### Data Analysis

#### Data Reduction

MEPs were analyzed offline using custom written Matlab scripts (MATLAB R2011b, The MathWorks, Natick, Massachusetts, U.S.A.). EMG data were demeaned, filtered (4th order Butterworth, 10–500 Hz), and signal averaged over 20 trials per condition. SICI was quantified by calculating 1 – the ratio of conditioned MEP_area_/unconditioned MEP_area_ (C/U ratio) where positive values indicate inhibition and negative values indicate disinhibition (51). EMG during the 100 ms period preceding the stimulus was analyzed offline to determine the magnitude of background EMG activity during muscle contraction (46). The ratio of S2 MEP size to background EMG activity (MEP/EMG) was also calculated.

#### Statistical Analysis

To address our primary question, whether IH SICI is normalized in chronic stroke, data analysis focused on IH SICI with participants grouped by lesion location (i.e., cortical, sub-cortical). Statistical analyses were performed using IBM SPSS Statistics 22 (SPSS Inc., Chicago, IL, USA). Data were found to meet the normality assumption using the Kolmogorov-Smirnov test.

For stroke survivors, mixed design [Hemisphere(2) × Task(3) × Lesion location(2)] ANOVA, with repeated measures on Hemisphere and Task, was used to analyze SICI and S2 MEP size. Background EMG and MEP/EMG were analyzed using similar mixed design [Hemisphere(2) × Task(2) × Lesion location(2)] ANOVA, with repeated measures on Hemisphere and Task. Subsequently, each hemisphere of stroke survivors was compared separately against the control group using mixed design [Group(2) × Task(3)] ANOVA with repeated measures on task. Within each hemisphere, comparisons were performed between cortical stroke, subcortical stroke, and controls using mixed design [Group(3) × Task(3)] ANOVA with repeated measures on task. All data met the Sphericity assumption, which was tested using Mauchly's test. Based on suggestions of current statistical literature, *post-hoc* comparisons, with Bonferroni correction for multiple comparisons, were conducted regardless of F-test results (52–55). Effect sizes (Cohen's d for two group comparisons, where Effect sizes = 0.2 are considered small, 0.5 medium, and ≥0.8 large; or Cohen's f for comparisons between more than two groups, where 0.1 is considered small, 0.25 medium and ≥0.4 large) (56) were also computed for all multiple comparisons.

RMT, AMT and ISI were each compared between hemispheres in stroke survivors using paired *t*-tests; each hemisphere was then compared with the control group using independent *t*-tests. Mixed design [Group(3) × Task(3)] ANOVA was used to analyze neuronavigation target errors in both hemispheres of stroke survivors and the control group, with repeated measures on task.

Pearson correlations were used to investigate the relationship between motor function scores and SICI, S2 MEP size, background EMG and MEP/EMG during each task. Statistical significance was established at *P* < 0.05.

## Results

Neuronavigation displacement and angle errors both fell within the target range (<3 mm and <5°). Displacement error was consistent between groups, hemispheres, and across tasks (*P*'s > 0.05). Angle error was somewhat greater during B&B compared to rest and grip (*P*'s < 0.02) without differences between groups or hemispheres (Table 3). No significant differences in RMT, AMT, or ISI were revealed between hemispheres or groups (*P*'s > 0.05).

**Table 3:** Neuronavigation target error.

	**Displacement error (mm)**			**Angle error (**^****°****^**)**		
	**Rest**	**Grip**	**B and B**	**Rest**	**Grip**	**B and B**
IH	1.49 (±0.64)	1.48 (±0.51)	1.86 (±0.77)	2.64 (±1.40)	2.71 (±1.25)	3.76 (±1.51)^*^
CH	1.50 (±0.39)	1.59 (±0.38)	1.81 (±0.56)	2.59 (±1.07)	2.44 (±0.87)	3.36 (±1.52)^*^
Controls	1.37 (±0.89)	1.43 (±0.72)	1.56 (±0.63)	2.61 (±1.00)	2.43 (±1.02)	3.19 (±1.59)^*^

Data presented are mean(±SD).

* *indicates significant between-task differences (P < 0.05). Displacement error was minimal (<3 mm) and consistent between groups, hemispheres and across tasks (P > 0.05). Angle error was somewhat greater during B and B compared to rest and grip (P's < 0.02), but within our targeted range (<5°); no group or hemisphere differences were revealed. CH, contralesional hemisphere; IH, ipsilesional hemisphere; B and B, box and blocks task*.

Group means for ISI, S2 MEP size, background EMG, and MEP/EMG are reported in Table 4. These three parameters were evaluated for significant differences across tasks to determine whether variations in general motor excitability or MEP size, specifically, influence SICI. Responses were generally consistent between hemispheres and groups across tasks. No significant differences were revealed between hemispheres, or between cortical stroke, subcortical stroke and healthy controls during any task (*P*'s > 0.05). Furthermore, in stroke survivors IH S2 MEP size, mean prestimulus EMG, and MEP/EMG during grip and B&B were not significantly correlated with clinical severity (e.g., UE FMA and BBT) (*P*'s > 0.05). These results indicate variations in background EMG or MEP size are not likely confounding factors contributing to differences in SICI across tasks.

**Table 4:** ISI, S2 MEP size, prestimulus EMG activities, and MEP/EMG ratio.

	**ISI (ms)**	**S2 MEP size (μV)**			**Mean prestimulus EMG acitvity (μV)**		**MEP/EMG**	
		**Rest**	**Grip**	**B and B**	**Grip**	**B and B**	**Grip**	**B and B**
IH	3.25 (±0.91)	557.87 (±487.36)	548.73 (±327.20)	636.35 (±4014.51)	36.99 (±27.71)	37.24 (±37.31)	26.67 (±25.90)	24.48 (±18.59)
CH	3.06 (±1.17)	587.36 (±383.36)	747.48 (±576.44)	561.53 (±349.91)	71.08 (±58.68)	34.09 (±13.66)	16.86 (±20.44)	17.33 (±9.78)
Controls	2.95 (±0.64)	555.92 (±390.23)	464.63 (±323.37)	775.18 (±294.60)	68.27 (±39.10)	27.42 (±7.42)	10.02 (±12.25)	26.69 (±10.49)

*Data presented are mean (±SD). All parameters were generally consistent across hemispheres and groups in each task. ISI, interstimulus interval; MEP/EMG, the ratio of S2 MEP size to prestimulus EMG; CH, contralesional hemisphere; IH, ipsilesional hemisphere; B and B, box and blocks task*.

### SICI by Hemisphere

**Figure 1:**
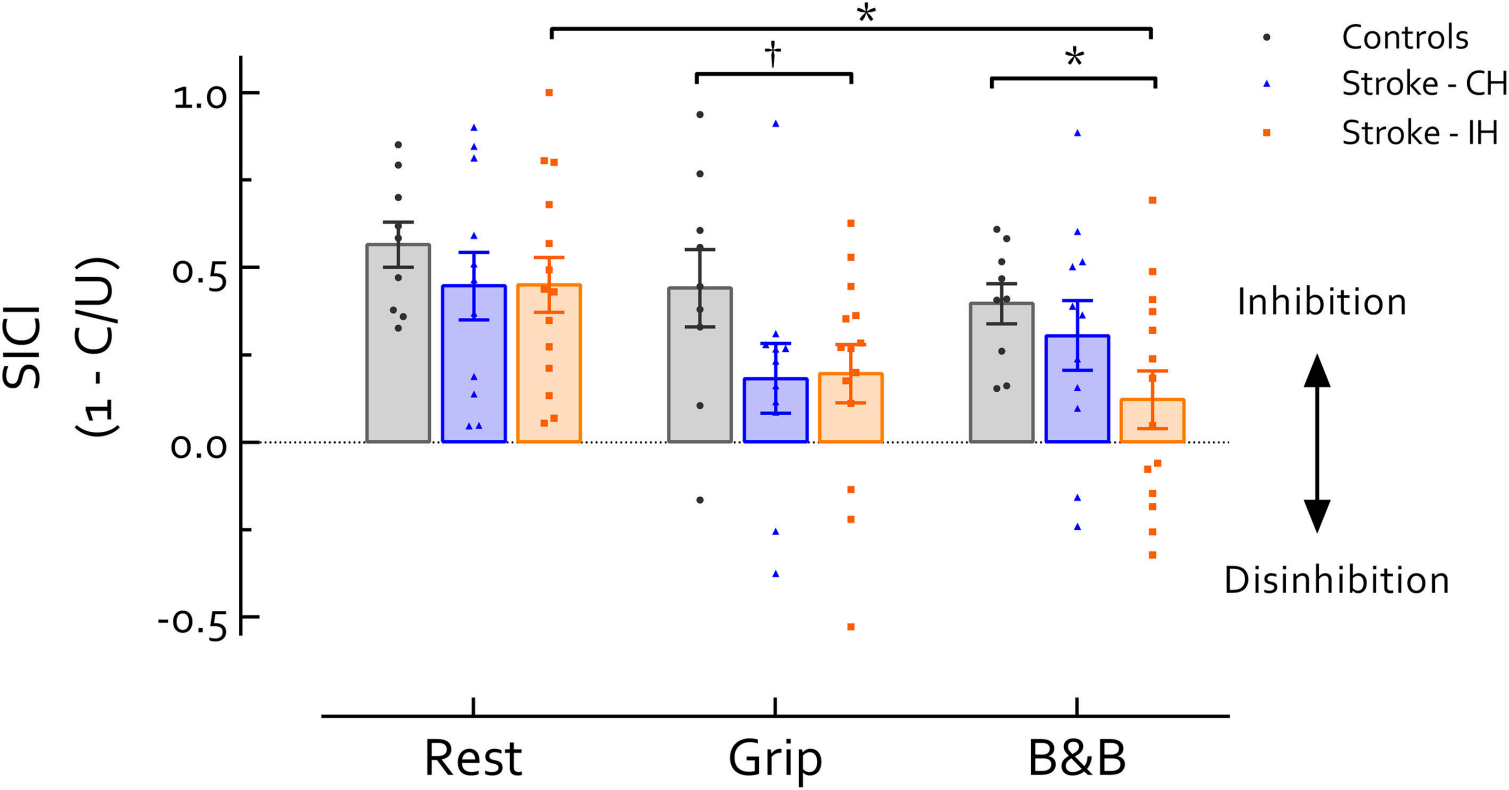
Short intracortical inhibition (SICI) across tasks and hemispheres. Data presented are group mean ±SEM. ^*^ indicates significant differences (*P* < 0.05). indicates differences approaching statistical significance (*P* < 0.1). No significant difference was revealed between IH and CH in stroke survivors. During grip and B and B, IH SICI was reduced compared with healthy controls (*P* = 0.094 during grip and 0.025 during B and B). Additionally, IH SICI was significantly reduced during B and B compared with rest (*P* = 0.028). In CH or healthy controls no difference in SICI was revealed across tasks. Of note, background EMG activity, S2 MEP size, and MEP/EMG were not significantly different between controls and Stroke IH during either grip or B and B, reducing likelihood that differences in motor excitability contribute to differences in SICI between controls and IH in stroke. CH, contralesional hemisphere; IH, ipsilesional hemisphere; B and B, box and blocks task; MEP, motor-evoked potential. MEP/EMG refers to the ratio of S2 (unconditioned) MEP size to mean prestimulus EMG activity.

No significant differences were revealed between hemispheres in stroke survivors, or between the CH and controls in any task (*P*'s > 0.05). Comparison between the IH and the control group revealed significant main effects of Task [*F*_(2, 42)_ = 3.86, *P* = 0.029] and Group [*F*_(1, 21)_ = 11.65, *P* = 0.003] (Figure 1). Follow up comparisons revealed that during active tasks, SICI in the IH was lower than controls; this difference approached significance during grip (*P* = 0.094, *d* = 0.76) and reached significance during B&B (*P* = 0.025, *d* = 1.09). At rest, there was no significant difference in SICI between IH and controls (*P* > 0.05, *d* = 0.46). In addition, IH SICI_B&B_ was significantly reduced compared with SICI_rest_ (*P* = 0.028, *d* = 0.63). In the control group, however, no differences in SICI were revealed across tasks (*P* > 0.05, *f* = 0.39). In the CH, although there was a significant main effect of Task (F_(2, 20)_ = 3.91, *P* = 0.37, *f* = 0.62), no significant differences in SICI across tasks were revealed in *post-hoc* comparisons (*P*'s > 0.05, *d*'s < 0.7) (Figure 1).

### SICI by Lesion Location

**Figure 2:**
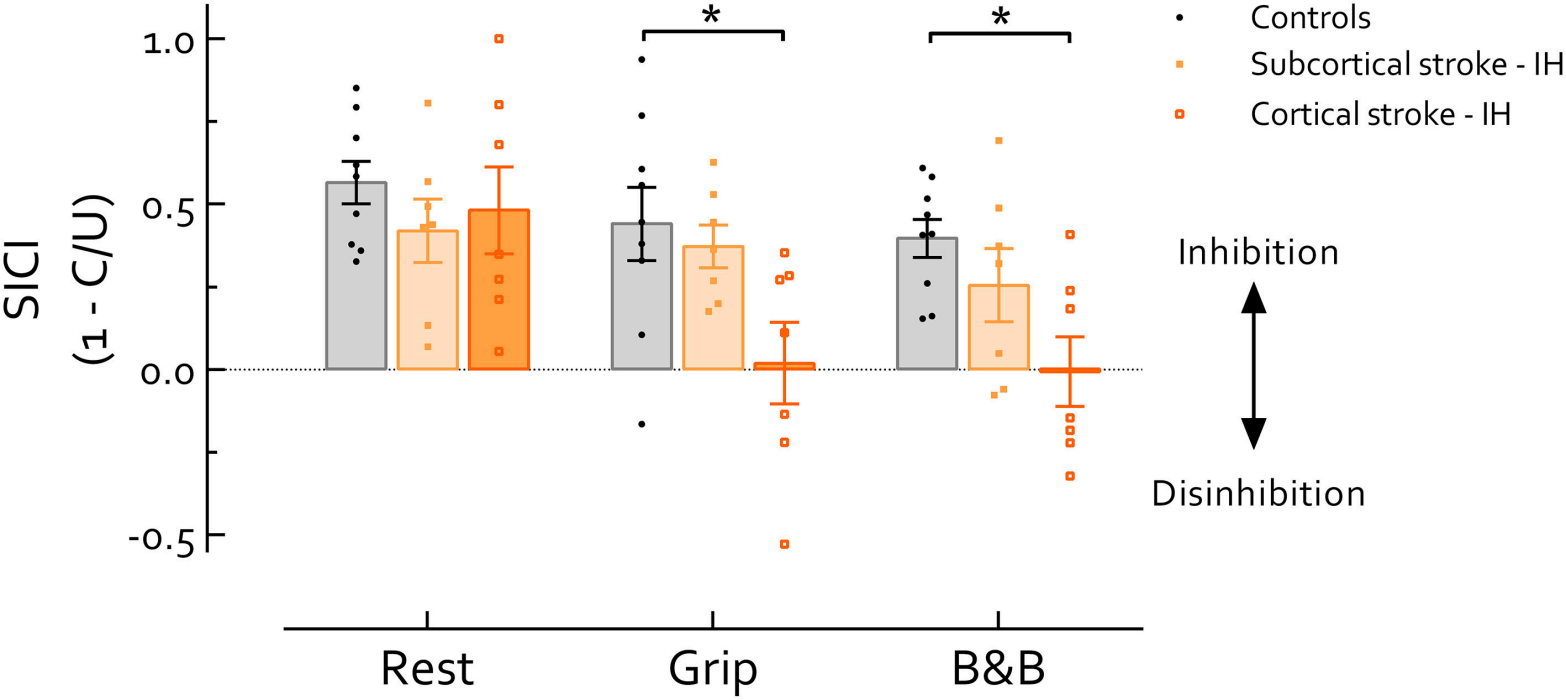
Short intracortical inhibition (SICI) in the IH in stroke survivors and healthy controls. Data presented are group mean ±SEM. ^*^ indicates significant difference (*P* < 0.05). During grip and B and B, SICI was significantly reduced in cortical stroke compared with healthy controls (*P*'s = 0.03 and 0.012, respectively). Across tasks, SICI in cortical stroke was significantly reduced during grip and B and B compared with rest (*P*'s = 0.04 and 0.017, respectively). Of note, background EMG activity, S2 MEP size, and MEP/EMG were not significantly different among groups during either grip or B and B, reducing likelihood that differences in motor excitability contribute to group-differences in SICI. IH, ipsilesional hemisphere; B and B, box and blocks task; MEP, motor-evoked potential. MEP/EMG refers to the ratio of S2 (unconditioned) MEP size to mean prestimulus EMG activity.

Our primary analysis, comparison between IH of cortical and subcortical stroke and healthy controls (Figure 2), revealed main effects of Task [*F*_(2, 40)_ = 5.19, *P* = 0.01] and Group [*F*_(2, 20)_ = 12.04, *P* < 0.001]. Follow up comparisons revealed that SICI was significantly lower during grip and B&B in cortical stroke than controls (*P*'s = 0.03 and 0.012, *d*'s = 1.23 and 1.74, respectively); while at rest, no difference in SICI was revealed across groups (*P* > 0.05, *f* = 0.23). In cortical stroke, SICI_grip_ (*P* = 0.04, *d* = 0.77) and SICI_B&B_ (*P* = 0.017, *d* = 0.82) were significantly reduced compared with SICI_rest_. In subcortical stroke, no differences in SICI were revealed across tasks (*P* > 0.05, *f* = 0.37) (Figure 2).

### Relationship Between Motor Performance and SICI

**Figure 3:**
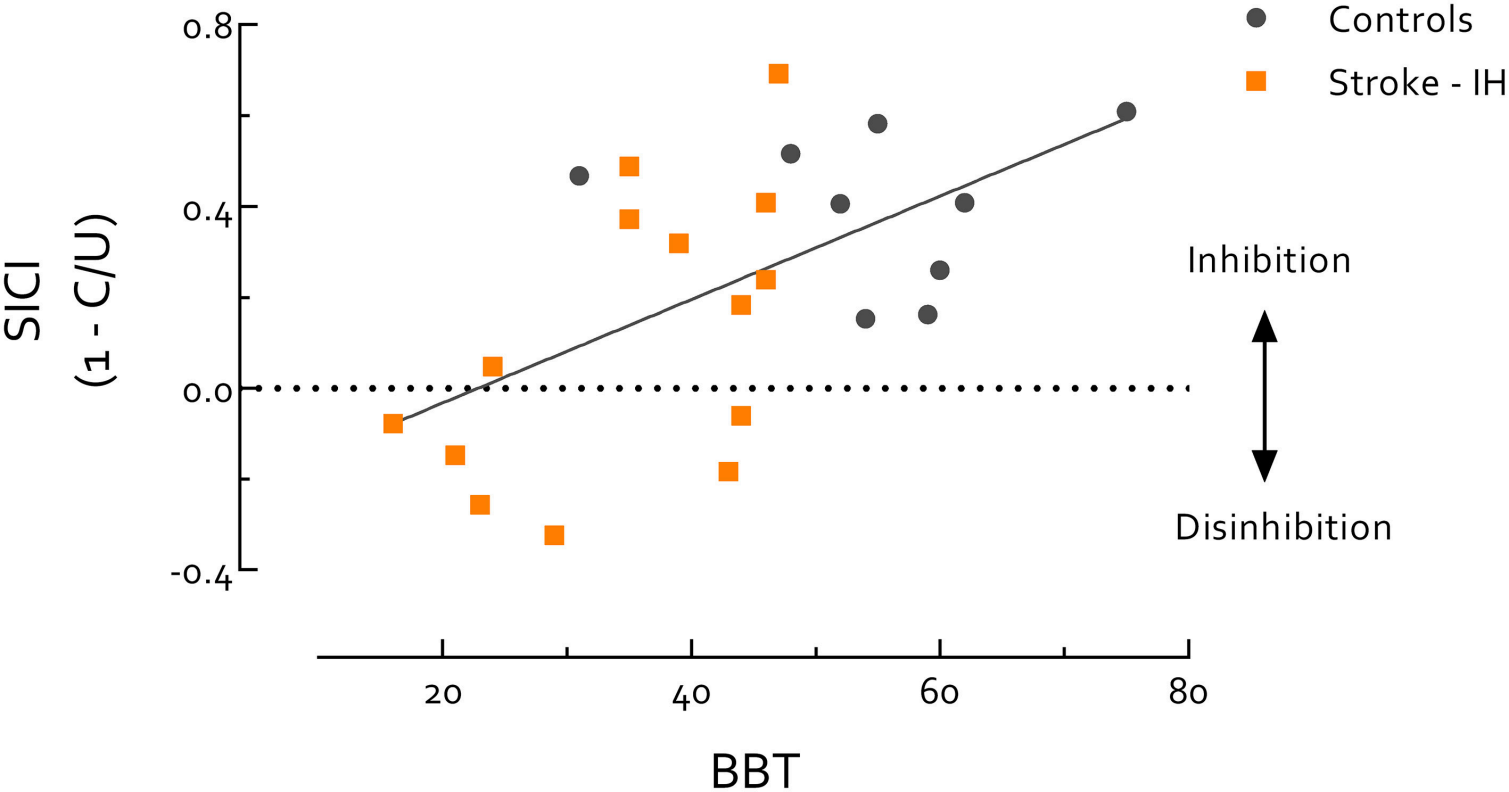
Short intracortical inhibition (SICI) measured during box and blocks task (B and B) is significantly correlated with Box and Blocks Test (BBT) score. Correlation includes all participants, stroke survivors and healthy controls, and all participants were evaluated on the same continuum. Most healthy controls showed better performance in BBT than stroke survivors, but there is also a region of overlap in BBT between healthy controls and stroke survivors. Individuals with better motor performance (i.e., healthy controls or high-functioning stroke survivors) tend to have more SICI during B and B, while individuals with poor motor performance (i.e., low-functioning stroke survivors) tend to have reduced SICI in IH during B and B (*r* = 0.57, *P* = 0.005). This result indicates that SICI-related inhibitory circuits may play an active role in coordinated motor activity. Of note, background EMG activity, S2 MEP size, and MEP/EMG during B and B were not significantly correlated with BBT score, therefore do not contribute to the correlation between SICI and BBT score. IH, ipsilesional hemisphere; MEP, motor-evoked potential. MEP/EMG refers to the ratio of S2 (unconditioned) MEP size to mean prestimulus EMG activity.

**Figure 4:**
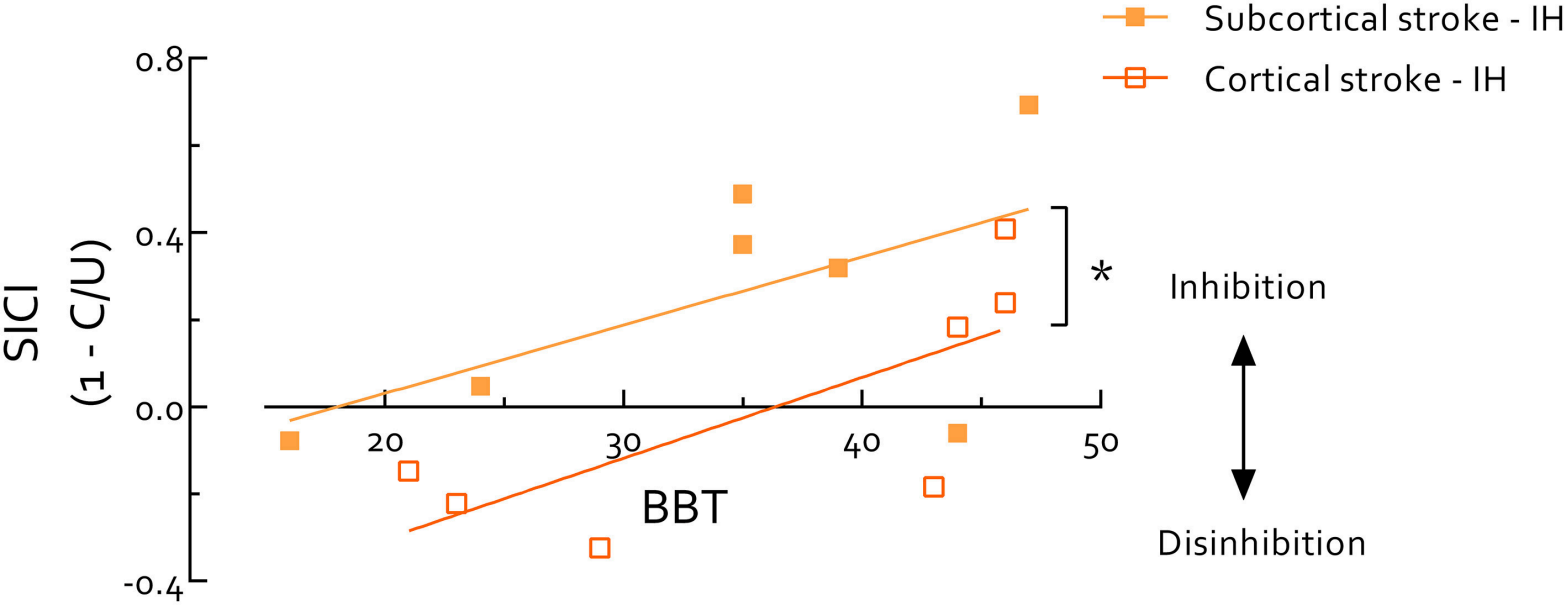
Short intracortical inhibition (SICI) in ipsilesional hemisphere measured during performance of box and blocks (B and B) correlates with box and blocks test (BBT) score in the paretic hand in stroke survivors. ^*^indicates significant difference (*P* < 0.05). Overall correlation reveals an association between SICI during movement and motor function (*r* = 0.56, *P* = 0.039). BBT scores span a similar range between cortical and subcortical stroke survivors, but the intercept is significantly lower (*P* = 0.01) in cortical stroke indicating systematically reduced SICI during B and B compared to subcortical stroke. Of note, background EMG activity, S2 MEP size, and MEP/EMG during B and B were not significantly correlated with BBT score, therefore do not contribute to the correlation between SICI and BBT score. IH, ipsilesional hemisphere; MEP, motor-evoked potential. MEP/EMG refers to the ratio of S2 (unconditioned) MEP size to mean prestimulus EMG activity.

Paretic hand BBT scores were highly correlated with the UE-FMA in all stroke survivors (*r* = 0.95, *P* < 0.0001). Thus, due to the known ceiling effect of the FMA (57, 58), we used the BBT to evaluate stroke survivors and controls on the same continuum of motor function. Using data from stroke survivors' IH and healthy controls we found SICI_B&B_ was positively correlated with BBT score (*r* = 0.57, *P* = 0.005) (Figure 3). Within only stroke survivors, IH SICI_B&B_ was also positively correlated with paretic hand BBT score (*r* = 0.56, *P* = 0.039). When the stroke group was separated by lesion location, the intercept of this relationship was significantly higher in cortical than subcortical stroke (*P* = 0.01) (Figure 4). IH SICI_B&B_ was also positively correlated with FMA (*r* = 0.56, *P* = 0.037) (not illustrated). However, no significant correlations were revealed between IH SICI_rest_ or SICI_grip_ and behavioral parameters (i.e., paretic hand BBT score or FMA) and SICI in the CH or in healthy controls.

## Discussion

To our knowledge, this is the first study to measure SICI during motor activity in stroke survivors. Our primary findings are: (1) when measured at rest, IH SICI appears to be normalized in chronic stroke; but (2) when measured during motor activity, IH SICI is reduced, reflecting motor disinhibition, especially following cortical stroke or in the presence of severe motor impairment; (3) when S1 intensity is adjusted to induce maximal inhibition, SICI_rest_ and SICI_active_ are similar in healthy individuals.

### SICI Measured at Rest

#### IH SICI_rest_ Appears to be Normalized in Chronic Stroke

Consistent with previous studies reporting normalization of IH SICI_rest_ by 6 months post-stroke (27, 28), we observed the magnitude of IH SICI_rest_ was similar between age-matched healthy controls, cortical, and subcortical stroke survivors. Furthermore, no differences were revealed between cortical and subcortical stroke in SICI_rest_.

CH SICI_rest_ also appeared to be similar to healthy controls. Results regarding CH SICI_rest_ in chronic stroke remain inconsistent in the current literature. Similar to our findings, Shimizu et al. (30) reported that CH SICI returns to normal within 6 months following both cortical and subcortical stroke. However, Honaga et al. (28) reported that CH SICI remains reduced 6 months following cortical, but returns to normal following subcortical stroke. Dissimilarities between Honaga et al.'s (28) results and ours may stem from differences in chronicity of stroke survivors studied [~2 years (28) vs. ~6 years, present study]. It is possible that if CH SICI_rest_ is normalized, it may occur over a wider time span following stroke than previously suggested.

#### Confounding Influences on SICI, Measured at Rest

While we observed “SICI non-responders” in both stroke and control groups, it remains unclear whether absence of SICI reflects the range of normal physiological variation or represents a pathological phenomenon (47, 48). Important to the current study, however, inability to induce SICI_rest_ may influence the function of SICI-related inhibitory circuits during motor activity, making it difficult to compare SICI_active_ between “SICI non-responders” and individuals exhibiting normal SICI_rest_. Therefore, we excluded the hemispheres (28, 32, 59) in which we were unable to induce SICI_rest_ at any ISI. Factors influencing the magnitude and presence of SICI_rest_ are complex and explanation for occurrence of four “SICI non-responders” among the larger sample is beyond the scope of the present study. However, methods to induce SICI were consistent across all participants, suggesting individual physiologic differences contribute to the phenomenon of “SICI non-responders.”

Variability in MEP size influences the presence and magnitude of SICI (60) and is much higher at rest than during voluntary muscle contraction (33) likely reflecting a fluctuating physiological state at rest (61). MEP size, and by extension SICI, are influenced by many physiological factors including: attention (62), speech (61), motor imagery (63–66) and movement observation (67–69), which are difficult to control when measuring neurophysiological responses at rest. Recognized inconsistencies in the existing SICI literature may stem from increased variability when SICI is measured at rest. Measuring SICI during controlled motor activity may stabilize the level of background neural drive across individuals, thereby reducing variability in SICI, and improving the likelihood of detecting genuine group or task differences.

While previous studies have suggested that IH SICI returns to normal in the chronic phase of stroke recovery (27, 28), this conclusion is based on studies in which SICI was measured at rest. Such results contribute to the impression that GABAergic inhibitory circuit function is ultimately normalized after stroke. However, since GABAergic inhibitory circuits have been found to contribute to selective muscle activation during coordinated motor tasks (2–5), SICI evoked at rest may not be the optimal methodology to assess functional recovery of this inhibitory network in chronic stroke.

### SICI During Motor Activity

Compared with healthy controls, IH SICI_active_ was reduced in chronic stroke. Importantly, S2 MEP size and background EMG during motor tasks were similar across groups, thus are unlikely to contribute to this group difference in SICI. Additionally, we found reduced IH SICI_active_ revealed significant influences of lesion location and motor function.

#### Lesion Location

Whether lesion location influences IH SICI_rest_ remains controversial. We found both IH SICI_grip_ and SICI_B&B_ were reduced in chronic stroke compared with healthy controls, especially following cortical stroke. Because SICI is suggested to be of cortical origin (23), it is reasonable to speculate that SICI-related inhibitory circuits are more likely to be disrupted in cortical than subcortical stroke. Indeed, it has been reported that SICI is more affected in the early phase following cortical vs. subcortical stroke (27, 30), but it has also been reported that lesion location does not influence IH SICI_rest_ more than 1 month post-stroke (8, 10, 26, 28). In the chronic phase however, differences between cortical and subcortical stroke are less clear, particularly whether and how GABAergic inhibitory circuits are normalized over time and how they function during motor activity. Activity of SICI-related circuits is argued to prevent unwanted muscle activation (2, 3) contributing to production of fractionated activity in intrinsic hand muscles (4–6). Therefore, greater activity in these circuits can be expected during both precision grip and B&B tasks. Although lesion location may not influence SICI_rest_ in the chronic phase following stroke, it appears to influence SICI_active_.

During grip, participants were asked to produce and maintain a stable, submaximal force level with visual feedback. SICI-related inhibitory circuits may contribute to this type of motor activity by inhibiting excessive muscle activation. During B&B, TMS was delivered concurrently with achieving maximal finger-thumb aperture prior to grasping a block. At this point in movement preparation, the velocity and direction of finger movements are carefully controlled, thus likely involve inhibitory activity to coordinate finger movements. Our observation of reduced SICI_active_ in cortical stroke suggests dysfunction of inhibitory GABA circuits during this coordinated motor activity. Although apparently normal at rest, our findings indicate the function of inhibitory GABAergic circuits may not be fully recovered following stroke, especially following cortical stroke.

#### Motor Function

Across all participants, our results revealed a positive correlation between SICI_B&B_ and motor function scores, implicating SICI_B&B_ as a functional correlate of motor performance. In lower-functioning stroke survivors, SICI_B&B_ is markedly reduced, or wholly deficient. Of note, no significant correlation was revealed between motor function and SICI_rest_ or SICI_grip_. There are two possible explanations for the correlation between paretic arm motor function and SICI_B&B_: greater impairment of SICI-related brain circuits in lower-functioning individuals, or differences in the relative muscle contraction level during B&B.

##### Relative contraction level

Relative contraction level is an important consideration when measuring SICI_active_ and a possible explanation for reduced SICI_B&B_ observed in low functioning individuals. While the absolute force requirement of B&B is constant and the grip force required for lifting a light object should be similar for stroke survivors and healthy adults (70), it is possible that a higher relative muscle contraction level was required during B&B in low-functioning individuals. The importance of this distinction is that SICI tends to be decreased at contraction levels >10% MVC (46) due to: reduced GABAergic inhibition, superimposition of concurrent facilitation from increased spinal motoneuron excitability (46, 71), or recruitment of short intracortical facilitation (SICF) (46), leading to less net inhibition. The confounding influence of SICF can be eliminated by setting S1 at 70% AMT (46) as was done in the current study. Increased spinal motoneuron excitability, as occurs with higher background contraction force, causes I-wave facilitation (72), specifically observed as increased I1 and reduced I3 contributions to MEPs (71, 72). During higher level muscle contraction (>10–15% MVC) later I-waves (i.e., I3) are not required to generate a test MEP size of 1 mV (46, 71, 72). Importantly, SICI acts mainly on the I3 wave with little influence on the I1 wave (71, 73, 74). Thus, less SICI is observed at higher contraction levels because there are fewer I3 waves to suppress (46).

Additionally, it has been suggested that when S1 = 70% AMT, low level muscle contraction (i.e., 0–10% MVC) does not influence SICI (46). The mass of each wooden block is ~10 g (~0.1 N) translating to a minimum grip force requirement of ~0.1 N. The magnitude of safety margin for lifting a light object is considered to be similar between stroke and healthy adults (70). Therefore, although not directly measured here, grip force is usually low (0.5–1 N) when grasping and lifting a small wooden block (70, 75, 76), well <10% MVC in most, if not all, participants studied here. Furthermore, our results revealed no significant correlations between motor function and S2 MEP size, background EMG, or MEP/EMG during B&B. Taken together, it is unlikely that SICI was strongly influenced by the relative contraction levels during B&B. Therefore, our observed correlation between SICI_B&B_ and motor function is more likely due to impaired GABAergic inhibition in lower-functioning individuals.

##### Effects of motor impairment

As mentioned above, while SICI_active_ has not been measured in stroke survivors, correlations between SICI_rest_ and motor impairment post-stroke have been previously investigated producing conflicting results (13, 28, 29). Honaga et al. (28) observed that chronic stroke survivors who exhibit more SICI_rest_ (i.e., more inhibition) tend to have better paretic arm motor function. Recently, Ferreiro de Andrade et al. (29) reported an opposite correlation, while still other studies report no correlation between SICI and motor function in chronic stroke survivors (8, 13, 27). Such inconsistent results may indicate that SICI_rest_ does not accurately reflect the function of GABAergic inhibitory circuits as they relate to motor control. Similarly, our SICI_rest_ results did not differentiate between control and stroke, or between high and low functioning stroke survivors. In contrast, SICI_active_ clearly differentiated between healthy controls and stroke survivors. While maintenance of stable, low level grip force may involve activity of GABAergic inhibitory circuits, power grip itself does not require individuated finger movements or place high demands on selective muscle activation. Consistent with this premise, our data show that SICI_grip_ was generally similar across stroke survivors, regardless of functional level. The B&B task, however, involves manual dexterity and finger coordination, arguably the types of movements in which GABAergic inhibitory circuits are actively involved. The robust association demonstrated between motor function scores and SICI_B&B_, included all participants—healthy and stroke regardless of lesion location—strongly suggesting both a role of GABA_A_-mediated inhibition in motor control and a functional consequence of deficient SICI in low functioning stroke survivors.

### SICI Across Tasks With Adjusted Conditioning Stimulus

We adjusted conditioning stimulus (i.e., S1) intensity between rest and active motor tasks to induce maximal SICI in each condition (4, 23, 46, 77–79). Our results contrast with most other studies that use the same S1 intensity across tasks and show reduced SICI during muscle contraction compared with rest (4, 77–79). Instead, our results reveal similar SICI_rest_ and SICI_active_ in healthy adults.

**Figure 5:**
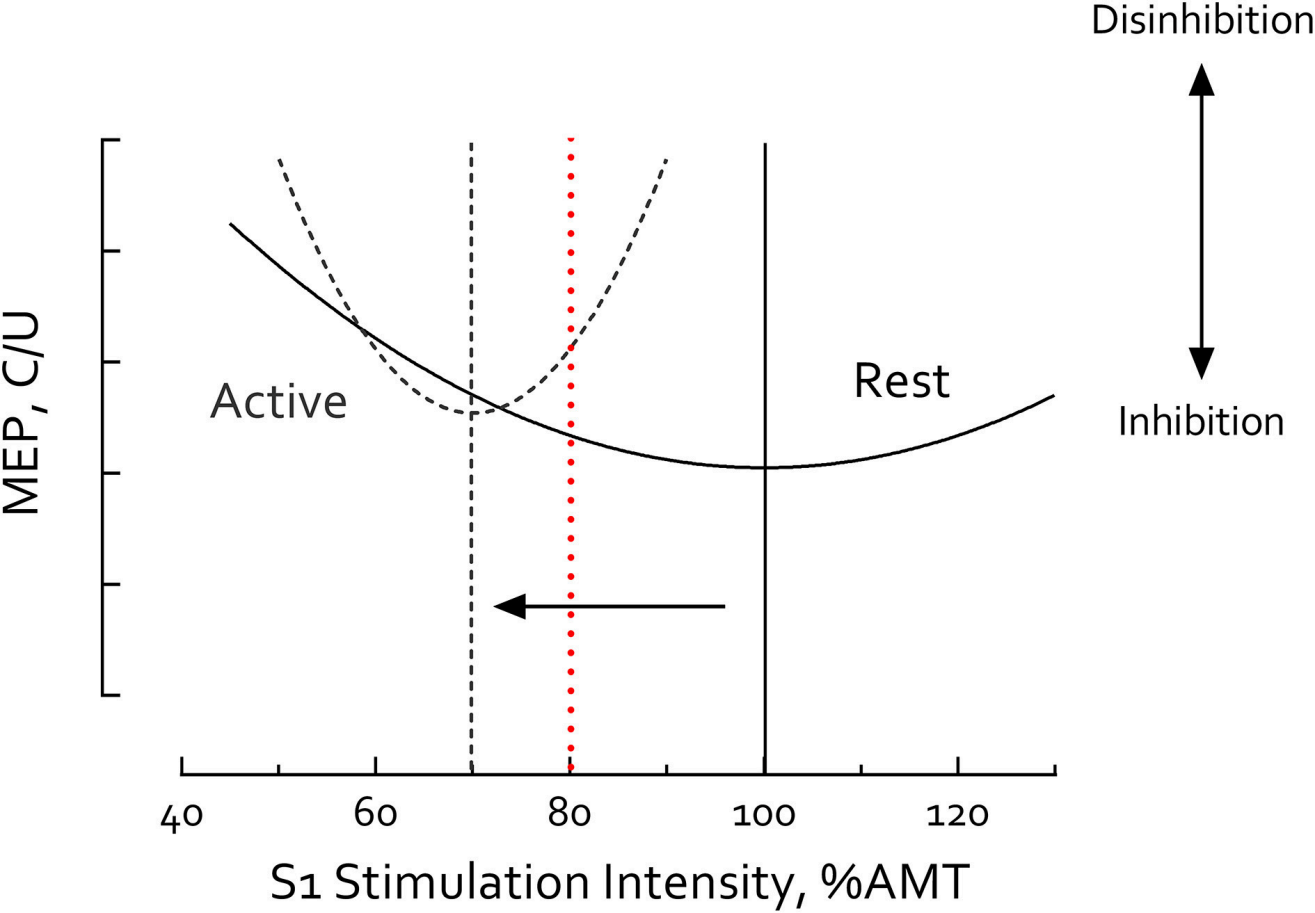
S1 Intensity-Short intracortical inhibition (SICI) relationship differs between rest and motor activity. Illustrative curves constructed using results compiled from published data obtained in healthy adults (23, 46, 80, 81). S1 Intensity-SICI curve is left-shifted during motor activity (dashed line) compared to rest (solid line). Vertical lines mark the minima of each curve. At rest, the low point of the U-shaped curve (i.e., the S1 intensity inducing maximal inhibition) falls ~100% active motor threshold (AMT) or 80% resting motor threshold (RMT) (23, 25, 46, 80, 81); but during muscle contraction, the curve is left-shifted with the low point falling ~70% AMT (arrow) (46). Using S1 = 80% RMT at rest and 70% AMT during active tasks the current study revealed no significant differences between SICI_rest_ and SICI_active_ in healthy controls suggesting these parameters induced maximal SICI in both conditions. Other studies have used the same S1 intensity (≥80% AMT) in both rest and active conditions (e.g., 80% AMT, red dashed line) (4, 77–79). In such cases, observation of reduced SICI during muscle contraction is not surprising, due to comparisons at different points of the resting and active S1-SICI curves. MEP, motor-evoked potential. C/U refers to conditioned MEP/unconditioned MEP. Greater C/U ratio indicates less SICI and disinhibition.

It is well-recognized that the magnitude of SICI depends critically on S1 intensity (23, 42, 46, 80, 81). Variation of S1 intensity at a given S2 intensity typically reveals a *U*-shaped relationship in SICI magnitude (23, 42, 80, 81) (Figure 5) with the lowest point of this U-curve ascribed to increasing recruitment of inhibitory interneurons that contribute to SICI (81). While the mechanism responsible for the high end of this curve is less clear, it has been suggested that SICF-related brain circuits are recruited and superimpose with inhibition thus reducing SICI magnitude (24, 42, 81). S1 intensity has also been reported to have differential influences on SICI_rest_ and SICI_active_ (46, 82), producing different *U*-shaped curves between resting and active muscle contraction. At rest, the S1 intensity which induces maximal inhibition falls around 80% RMT (or 100% AMT) (23, 25, 46, 80, 81); but during muscle contraction, this curve is left-shifted with the low point falling at 70% AMT (46) (Figure 5). Our goal in the present study was to induce maximal SICI in each task which motivated the decision to vary S1 intensity between 80% RMT for the resting condition and 70% AMT during active motor tasks.

To our knowledge, maximal SICI—at the putative low point of the U-curves—has not previously been reported. Using this method, we observed no differences between SICI_rest_ and SICI_active_ in healthy individuals. This is a novel finding suggesting that maximal SICI is similar whether induced at rest or during motor activity. We acknowledge that most other studies use the same absolute S1 intensity during both rest and active muscle contraction and, consistent with Ortu et al. (46), report reduced SICI_active_ compared with SICI_rest_. Due to this methodological difference, it is not possible to directly compare modulation of SICI across tasks between ours and other studies. However, we posit that our experimental approach maximally engages GABAergic inhibitory circuits in each condition, revealing reduced SICI_active_ in the IH even in the hyper-chronic stage post-stroke. Because only one S1 intensity was tested in each task it remains unclear whether a horizontal or vertical shift in the U-curve caused the reduction in IH SICI_active_ post-stroke; further studies are needed to answer this question. Regardless, our data contrast with the literature suggesting that SICI normalizes as part of the natural history of motor recovery. Deficits in the function of inhibitory circuits remain in chronic stroke and likely affect task-dependent regulation of motor circuits during active task performance.

### Clinical Implications

Observation in animal models that blockage of extrasynaptic GABA_A_ receptors is related to increased cortical plasticity and functional recovery acutely following stroke (11) contributes to expectation that a similar reduction in GABAergic inhibition is critical to motor recovery in the early phase post-stroke in humans (7–10). As a result current views on stroke rehabilitation emphasize means to increase IH cortical excitability (i.e., NIBS, intensive paretic limb rehabilitation, etc.) in both acute and chronic stroke survivors (16–19). Effects of these rehabilitative interventions are, however, limited. Furthermore, only a sub-set of stroke survivors are able to benefit (21, 22).

Our findings implicate an important role for GABAergic intracortical inhibition in motor recovery, at least in the chronic phase post-stroke, and may explain why therapeutically increasing IH cortical excitability regardless of individual's baseline neurophysiological state does not always contribute to a beneficial functional outcome. Demonstration of a relationship between net cortical excitability and strength (83, 84) contributes to the ostensible premise that increased IH cortical excitability may be related to strength improvement following stroke (85, 86). However, performance of dexterous motor tasks requires activity of intracortical inhibitory circuits to gate, or shape, motor excitability in response to task demands. As a result, increased cortical excitability alone cannot be expected to improve dexterous motor function.

Despite findings in the extant literature (2–6, 13, 14), the importance of intracortical inhibitory circuits to motor recovery post-stroke remains under-appreciated in neurorehabilitation. This oversight is possibly due to the belief that SICI is normalized during the natural course of stroke recovery and therefore is not associated with motor function in chronic stroke survivors. Our results suggest that inconsistencies in the current SICI literature in stroke survivors likely result from insensitivity of SICI_rest_, and furthermore that SICI_active_ is more sensitive for revealing motor impairment post-stroke. By measuring maximal SICI during motor activities, our results reveal that activity of GABAergic brain circuits is not normalized, even in the hyper-chronic phase following stroke. Moreover, reduced GABAergic activity (e.g., disinhibition) in stroke survivors negatively impacts motor function. Inhibitory circuit function may therefore serve as a physiological biomarker of unfulfilled motor recovery in the chronic phase post-stroke.

### Limitations

We acknowledge limitations of the present study. Stimulations were delivered manually during B&B, at the point of maximum finger-thumb aperture during grasp preparation. In future work, an instrumented device to trigger stimulations in conjunction with a movement event could improve experimental consistency. While muscle fatigue may influence SICI (87, 88), tasks were tested in randomized order, therefore differences across tasks observed here are unlikely to result from fatigue. The sample size in this study is relatively small, but this limitation is mitigated somewhat by normal distribution of data and large effect sizes. We recommend future studies involve a larger number of stroke survivors in various phases of stroke recovery to confirm and extend our findings.

### Conclusion

In conclusion, this is the first study to measure SICI_active_ in stroke survivors. Although differences in SICI_rest_ were not revealed between chronic stroke survivors and healthy controls, IH SICI_active_ was reduced post-stroke and IH SICI_B&B_ was significantly associated with paretic arm motor function. Taken together, our results suggest that the functionality of GABAergic inhibitory networks remains altered, even in the chronic phase post-stroke, and may impede execution of dexterous motor tasks.

## Data Availability Statement

The datasets generated and analyzed for this study are available by request from the corresponding author.

## Author Contributions

CP and WT designed the experiment. SK, QD, and CP conducted the experiments. QD and SK reduced and analyzed data. QD, CP, and WT interpreted the data. QD, CP, WT, and SK wrote the manuscript.

### Conflict of Interest Statement

The authors declare that the research was conducted in the absence of any commercial or financial relationships that could be construed as a potential conflict of interest.

## Abbreviations

AMT: active motor threshold
B&B: box & blocks task
BBT: box and blocks test
CH: contralesional hemisphere
FDI: first dorsal interosseus
IH: ipsilesional hemisphere
ISI: interstimulus interval
MEP: motor-evoked potential
MEP/EMG: the ratio of S2 MEP size over the mean prestimulus EMG activity
MoCA: Montreal cognitive assessment
MVC: maximal voluntary contraction
NIBS: Non-invasive Brain Stimulation
RMT: resting motor threshold
SICF: short intracortical facilitation
SICI: short intracortical inhibition
TMS: transcranial magnetic stimulation
UE FMA: upper-extremity component of the Fugl-Meyer Motor Function Assessment.
